# Regulation of Translation by PKA Signaling Pathway

**DOI:** 10.3390/ijms27156789

**Published:** 2026-07-29

**Authors:** Lele Yang, Kun Hou, Huayu Qi

**Affiliations:** Center for Development and Regenerative Medicine, Guangdong Provincial Key Laboratory of Stem Cell and Regenerative Medicine, Guangdong-Hong Kong Joint Laboratory for Stem Cell and Regenerative Medicine, China-New Zealand Joint Laboratory on Biomedicine and Health, Guangzhou Institutes of Biomedicine and Health, Chinese Academy of Sciences, Guangzhou 510530, China; yang_lele@gibh.ac.cn (L.Y.); hou_kun@gibh.ac.cn (K.H.)

**Keywords:** PKA, regulatory subunit, AKAP, protein synthesis, spermatogenesis

## Abstract

Extracellular stimuli, including hormones, growth factors and nutrients in the milieu of cells often initiate intracellular changes via signaling pathways, of which the cyclic 5′, 3′-adnosine monophosphate (cAMP)-dependent protein kinase (PKA) signaling pathway is prototypical. Research in the past decades has demonstrated that PKA plays versatile roles during cell proliferation and differentiation, mainly through phosphorylating a plethora of protein substrates by its protein kinase activity. Studies using model systems including yeast, neurons and mammalian germ cells indicate that PKA functionality is regulated by not only the cell-type-specific expression of its regulatory and catalytic subunits, but also the spatiotemporal distribution of its binding proteins and secondary messengers. How PKA elicits its functional specificity in a spatiotemporal manner constitutes fundamental mechanisms that regulate development, aging and regeneration. In this review, we first summarize basic aspects that drive the functional diversity of PKA and then focus on the less studied regulatory roles of PKA during synthesis of cellular proteins, the functional units of the cell. Direct links between PKA signaling and protein synthesis machinery are yet to be fully characterized. We anticipate that research in this area, combining model systems and newly developed methodologies, will continue to deepen our understanding of animal development and the etiology of human diseases.

## 1. Introduction

The identity of a cell is defined by its intrinsic features, including its metabolic status and functional proteome, which are constantly shaped by extrinsic stimuli in the milieu of cells during animal development, growth, maintenance and aging. Extracellular signals, including growth factors, hormones and nutrients, relay their information through signal transduction pathways initiated by receptors, transporters or channels that reside on the plasma membrane. These cell surface proteins transmit effects of external stimuli (the primary messengers) through small molecules generated intracellularly (the secondary messengers), often via activating enzymes such as protein kinases in order to modulate the conformation and functionality of substrate proteins through post-translational modifications. Many kinase-mediated signal transduction pathways elicit such effects, among which the cyclic adenosine 3′, 5′-monophosphate (cAMP)-dependent protein kinase A (PKA) is one of the earliest known kinases. As its name implies, PKA is activated by cAMP (a secondary messenger) in response to hormones and nutrients among other stimuli. Activated PKA phosphorylates protein substrates that regulate cell proliferation, migration and differentiation. A wide variety of proteins, including ion channels, cytoskeletal proteins, transcription factors, as well as protein synthesis machineries, can be phosphorylated by PKA at conserved serine/threonine residues. Understanding how signaling networks interconnect environmental cues with cell autonomous features has been central to the studies of cell fate determination, differentiation, cellular growth, as well as etiology of human diseases. Research in the past decades has illustrated diverse functional roles of PKA and revealed many aspects of its working mechanisms, including its cross-talks with other signaling pathways that are sensitive to nutrient availability and energetic status of cells, such as the mechanistic target of Rapamycin complex (mTORC) and AMP-dependent kinase (AMPK) pathways, both of which are intricately associated with the regulation of cellular metabolic status and proteomic landscape. Although the regulation of protein synthesis was one of the first functional roles realized for PKA signaling, how activities of PKA interact with cellular metabolic status and protein synthesis remains to be fully understood. Direct links between PKA signaling and regulatory networks that maintain cellular proteostasis require further investigation, especially in the mammalian systems. In this review, we will first summarize basic aspects of PKA signaling, including its allosteric activation mechanism, the tissue-specific expression of its subunits, the modulatory roles of its anchoring proteins and its multiple effects on cells. We will then analyze the functional roles of PKA in regulating protein synthesis, using studies from baker’s yeast, neurons and mammalian germ cells as examples. Understanding how cells maintain proteostasis during animal development or when cells encounter unexpected perturbations, such as stress, will be facilitated by answering many questions anticipated in this area of research.

## 2. Signal Transduction Mediated by cAMP-Dependent Protein Kinase (PKA)

### 2.1. The Cyclic Nucleotide-Dependent Protein Kinase

**Figure 1:**
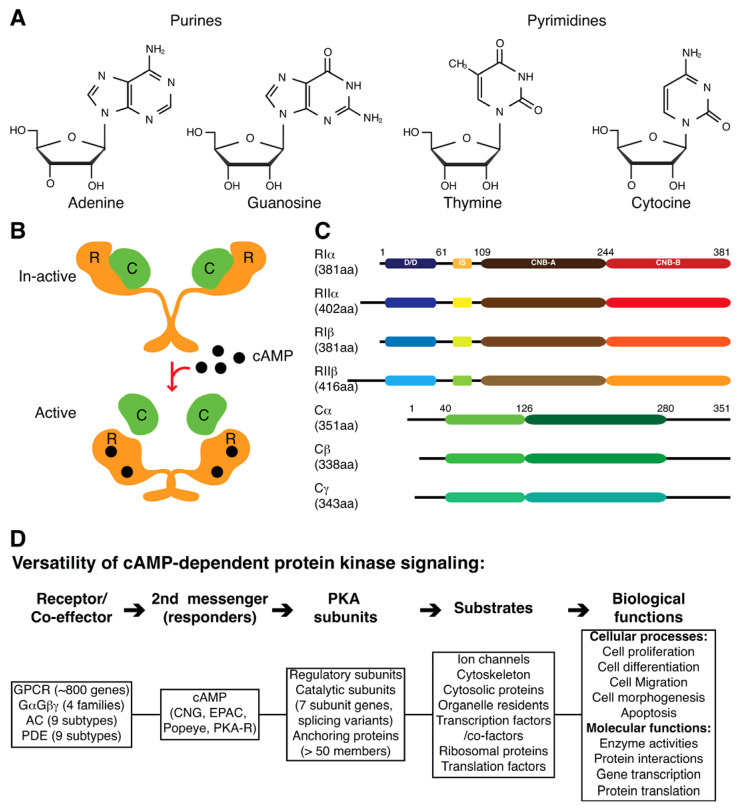
cAMP-dependent protein kinase (PKA) signaling pathway. (**A**): Nucleotides of purines and pyrimidines. Source: BioRender.com. (**B**): Allosteric activation of PKA holoenzyme. PKA holoenzyme is composed of a dimer of regulatory (R) subunits, holding two catalytic (C) subunits in inactive state. Each regulatory subunit can bind two molecules of cAMP and undergo conformational changes. This causes the release of the C subunits to phosphorylate substrates. (**C**): Protein domain features of PKA subunits. Isoforms of PKA subunits share conserved protein domain features, including the docking/dimerization (D/D) domain, inhibitory segment (IS) and cAMP-binding domains (CNB) in R subunits and the bi-lobal structure of C subunits. Numbers of amino acid residues are referenced in RIα and Cα. See also Table S1. (**D**): The versatility of PKA signaling can be caused by the combination of various components along the PKA signaling pathways, including multiple receptors, effectors, isoforms of subunits and protein substrates. Many of the PKA signaling components are expressed in a cell-type- and tissue-specific manner.

Nucleotides are the basic building blocks for nucleic acids that make up the genetic code. The metabolism of nucleotides not only interconnects basic nutritional resources with macromolecules such as carbohydrates, amino acids and lipids through metabolic intermediates but also generates signaling molecules in cells [1]. For example, the adenosine derivative adenosine tri-phosphate (ATP), the energy currency generated mainly through mitochondrial respiration in most cells, can also produce cyclic adenosine 3′, 5′-monophosphate (cAMP) as a secondary messenger (Figure 1A). This was first realized when an elevated level of cAMP was measured in mammalian cells treated with growth hormones epinephrine or glucagon [2]. Subsequent research found that the enzyme adenylyl cyclase (AC) catalyzes a cyclization reaction that forms hydrogen bonds between the α-phosphate and 3′- and 5′-carbon atoms of ribose conjugated to adenosine [3]. A similar reaction occurs when guanosine tri-phosphate (GTP) is catalyzed into cyclic guanosine 3′, 5′-monophosphate (cGMP) by guanylyl cyclase (GC) [4,5]. Other cyclic nucleotides, i.e., cUMP (cyclic uridine 3′, 5′-monophosphate) and cCMP (cyclic cytidine 3′, 5′-monophosphate), also exist in bacteria and mammalian cells, probably generated by the same family of nucleotide cyclases [6]. However, their functional roles as secondary messengers are yet to be understood. Cells have also evolved enzymes that counter the activity of cyclases in order to control the homeostasis of these secondary messengers. Phosphodiesterases (PDEs) that can specifically hydrolyze either cAMP or cGMP closely monitor their concentrations and regulate the duration of signaling activities in cells [7]. In eukaryotes, 11 PDE sub-families with more than 100 isoforms have been shown not only express in a cell- and tissue-type-specific manner, but also elicit subcellular localization and substrate specificities [8].

Hormones and glucagon were found to activate protein kinases along an increasing level of cAMP. Sutherland EM and others discovered that the cAMP could function as the key stimulator for protein kinases in different cell types, including skeletal muscle, adipocytes and neurons, during hormonal treatment [9,10]. The protein kinase whose activity depends on the cAMP was subsequently identified from rabbit skeletal muscle by Krebs’ lab using affinity purification method and named as the cAMP-dependent protein kinase (PKA) [11], which has a K_m_ of 60–100 nM for cAMP and requires the presence of ATP to phosphorylate substrates. PKA became the second protein kinase discovered after the phosphorylase kinase which itself is also a substrate of PKA [12]. These early works illustrated the importance of hormonal effects on tissue growth and metabolism and directed relevant mechanistic studies toward signal transduction pathways that are mediated by secondary messengers [13].

The composition and regulatory mode of the cAMP-dependent protein kinases were further revealed by biochemical analyses. Two cAMP-bound protein complexes, a few kilodalton apart, were separated using gel filtration and anion exchange chromatography from homogenates of rabbit skeletal muscle, namely the type I and type II PKA complexes [14,15]. Both PKA complexes contain a dimer of regulatory (R) subunits and two catalytic (C) subunits that together form an inactive tetrameric holoenzyme [16,17]. Each R subunit can bind two molecules of cAMP and undergoes a conformational change, which allows its dissociation from the C subunits that are now activated to phosphorylate substrates in the presence of ATP [18] (Figure 1B). This activation mode of enzyme has served as the paradigm for allosteric activation mechanisms not only for kinases but also for many other protein complexes [19,20,21]. The regulatory subunits of these PKA holoenzyme complexes are classified as RI (for PKA-I) and RII (for PKA-II), which can be further divided into α and β subtypes. There are currently seven known subunits of PKA, including RIα, RIβ, RIIα, RIIβ, Cα, Cβ and Cγ in mammals, each encoded by a different gene (Figure 1C). The tetrameric holoenzyme of PKA can be made up by a combination of different isoforms of α or β subunits. In addition, alternative splicing of the transcripts from genes encoding subunits could generate different variants, such as Cα1, Cα2 and Cα3, in different cells and tissues [22,23,24]. The diversity of PKA subunits suggests that different PKA subtypes may possess functional specificities which are supported by gene expression analyses and genetic studies in different animal models (see below) [25].

### 2.2. G Protein-Coupled Receptors (GPCRs) Transmit External Stimuli to PKA Signaling

Changes in intracellular cAMP concentration are responsible for the dynamic regulation of PKA activity. Research showed that intracellular cAMP is regulated in a spatiotemporal fashion through more than one mechanism [26]. The original findings showing the association of AC with particulate fractions of cell homogenates indicate that enzymes catalyzing cAMP production are localized at the plasma membrane [3,27,28]. Indeed, 10 members of the AC family have been found so far, all of which possess transmembrane domains and associate with cell surface receptors [28,29]. Following the discovery of the β-adrenergic receptor that responds to epinephrine [30], the membrane receptors that transmit signals from extrinsic stimuli to cAMP and thus PKA have increased rapidly into a large family of receptor proteins. These are proteins with seven-transmembrane domains flanked by N-terminal extracellular ligand recognition regions and C-terminal intracellular segments that interact with heterotrimeric GTPase complexes, often called G-protein coupled receptors (GPCRs) [31]. The intracellular segments of GPCRs also bind β-arrestins, which sometimes transduce signals through alternative pathways [32,33]. Upon binding of ligands, GPCRs trigger G_α_ subunit of the G protein complex to undergo conformational changes and dissociate from G_β/γ_ subunits in GTP-bound form. Both G_α_ and G_β/γ_ moieties of G protein complexes have been shown to elicit enzymatic activities towards downstream effectors [34] (Figure 1D). Depending on their downstream effects, G proteins are classified into sub-families of G_s_, G_i/o_, G_q/11_ and G_12/13_, of which G_s_ is the canonical activator of ACs that stimulates the generation of cAMP, whereas others could have negative effects in this regard [35,36]. Thus, depending on the subtypes of linked G proteins which are also expressed in a cell-type-specific manner, ligands of GPCRs convey differential effects inside cells [37,38].

GPCRs are currently the largest known family of receptor proteins that mediate a wide spectrum of extracellular stimuli, including hormones, growth factors, odorants, photons and even physical constrains [39]. The human genome contains about 800 genes encoding GPCRs according to the 7-transmembrane domains featured in their amino acid sequences. Many of them are still lacking known ligands, and thus are called orphan receptors [40]. On the other hand, some extracellular ligands that have been known to trigger intracellular cAMP changes and thus PKA activities are still missing bona fide corresponding GPCRs, including hormones and growth factors [41]. For example, both androgen and estrogen have been shown to regulate gene expression through nuclear receptors in germ cells; however, cell surface receptors that are part of these signaling pathways remain unidentified [42,43,44]. Combining bioinformatics and high-throughput screening methods, Forster et al. and others discovered a series of peptide ligands for previous orphan receptors [45,46]. A similar strategy could be applied to uncover many unknown functions of GPCR-PKA signaling pathways. The wide range of physiological functions facilitated by GPCRs makes them the most favorable targets for drug discovery and human disease intervention.

The regulation of intracellular levels of cAMP and subsequently PKA activity could also occur at the level of AC. About ten ACs divided into six sub-families have been discovered across different phyla, each with their own specific tissue expression patterns and different composition of subunits [28]. In addition to the canonical membrane AC, intracellular soluble AC (sAC) has also been reported in mammalian male germ cells and other cell types, which expands the localized fluctuation of cAMP levels to more defined intracellular domains [27,47]. In addition, cells could take up cAMP originating from neighboring cells through transporters or channels [48,49]. One mechanism that underlies cellular metabolic status and PKA signaling is that nutrients, such as glucose or lactate, that modulate the intracellular ATP levels and ultimately change available cAMP can directly interact with GPCRs [50]. In the same vein, a recent finding suggests that lactate regulates hepcidin expression during hepatic iron homeostasis by directly binding to sAC and increasing cAMP, which subsequently activates the PKA-Smad1/5/8 pathway [51]. Via differential substrate selectivity, tissue expression, subcellular localization and mode of activity regulation, PDEs create a local cAMP domain and monitor the duration of PKA signaling [52]. Nonetheless, the combinatorial effects of subtypes and cell-type-specific expression of GPCRs, G proteins, ACs and PDEs are all adding complexity as well as specificity of PKA signaling pathways [53,54,55]. The vast repertoire of upstream factors is partly the reason for the diversified physiological functions mediated by PKA.

### 2.3. Versatile Substrates of PKA Signal Transduction Pathway

The diversified functions of PKA also come from its large number of substrates. PKA catalyzes the transfer of a phosphate (PO_4_^−^) from ATP to the sidechain hydroxyl group of serine or threonine within consensus sequences of protein substrates. The canonical phosphorylation site of PKA contains RRXS/TX motif (X represents any amino acid), originally identified from yeast fructose-1,6-bisphosphotase [56]. Similar substrate specificities have then been investigated for mammalian PKA using both synthetic peptides and high-throughput methods [57,58]. With the completion of genomic sequencing in many organisms, including humans, hundreds of potential PKA substrates can be predicted based on the similarity of recognition motif, interaction and functional data of PKA, greatly accelerating the identification of novel roles of PKA signaling [59,60,61]. This led to the realization that PKA potentially regulates hundreds of proteins in cells, including ion channels, cytoskeletal proteins, resident proteins of organelles, transcription and translation factors, as well as metabolic enzymes, illustrating a wide spectrum of PKA activities. The monophosphate group from ATP carries negative charges which can influence the biochemical properties of substrate proteins and cause changes in protein interactions and enzyme activities. From this point of view, it is conceivable that PKA can effectively change the interaction of proteins and catalytic activities of metabolic enzymes upon phosphorylation of substrates. However, the functional roles of many of the predicted PKA substrates remain to be experimentally tested.

Effector proteins that regulate the proliferation and differentiation of cells under the control of PKA have been identified in most cell types in mammals. Many of these effectors are involved in regulating cellular activities such as gene expression, metabolism, cell cycle and morphogenesis, linking alterations of PKA activity to animal development, growth, aging and regeneration, as well as cancer and other metabolic and degenerative diseases [62,63,64,65]. Following the initial finding that protein phosphorylase kinase is a substrate of PKA, many metabolic enzymes and kinases are also found to be regulated by PKA, including endothelial nitric oxide synthase (eNOS), H^+^-ATPase and eukaryotic elongation factor-2 kinase (eEF2K) [66,67,68], indicating the close relationships among metabolism, translation and PKA activity. Proteins that are residents of organelles or in close vicinity phosphorylated by PKA facilitate the change in functional status of organelles so that they can be adapted to cellular events, including mitochondria [69,70]. PKA facilitates the regulation of cell cycle through phosphorylation of cyclins and CDKs. For example, in mammalian oocytes, PKA inhibits oocyte maturation by blocking meiosis progression at prophase-I [71,72]. This is mainly due to the regulation of the maturation-promoting factor (MPF), a CDK2/CyclinB complex that is usually rendered inactive by PKA phosphorylation [73,74]. In mammals, the surge of luteinizing hormone (LH) may induce the inhibition of AC, leading to a decrease in cAMP level and PKA activity, resulting in resumption of meiosis in oocytes (meiotic maturation) [75]. However, PKA-independent maturation has also been reported in Xenopus [76]. Cell migration and morphological changes are important events during development and tumorigenesis. Through cytoskeletal regulators, environmental cues trigger PKA activity to modulate the regulatory factors of cytoskeletal systems. It was first found that fibroblast rounded up once PKA activity was increased. The actin filaments were disassembled due to the phosphorylation of myosin light chain kinase (MLCK) by PKA during this reversible process [77]. PKA also phosphorylates actin filament regulator small G proteins including RhoA and Rac during cell migration and anchorage remodeling, processes that are also involved in tumorigenesis [78,79,80]. Planar cell polarity (PCP) complexes have also been shown to be substrates of PKA, indicating a role of the PKA in cellular migration and asymmetric cell division during development [81]. One of the most dramatic effects of PKA on cells is the modification of gene expression programs that affect numerous genes. This can be achieved at both transcriptional and post-transcriptional levels. Through phosphorylation of proteins involved in the DNA-binding activities, PKA changes the expression of numerous genes involved in metabolism, steroidogenesis and spermatogenesis in response to the external stimuli [82,83]. Epigenetic factors, transcriptional factors and their co-factors have all been implicated in the PKA signaling [84,85,86]. As protein synthesis takes up to one-third of cellular energy, it is not surprising that PKA was originally found to mediate the immediate protein translation response upon glucose depletion in yeast [87,88]. Much of the regulation of gene expression at the level of protein translation by PKA signaling has been investigated in model systems, including yeast, neurons and mammalian germ cells (see later).

## 3. Regulation of PKA Signal Transduction Pathway

The vast influences of PKA on cellular physiology require its activity to be regulated precisely in time and space. Although much has to be learned about how cells achieve the specificity and efficiency of PKA signaling, past studies have shown that the efficacy of PKA can be regulated at multiple levels. First, the structural differences in PKA subunits intrinsic to their molecular variations generate subtleties in their functional specificity; second, PKA subunits are expressed in tissue and cell-type-specific manners, limiting functional PKA subtypes to particular tissues; third, the subcellular localizations of PKA are rendered by specific anchoring proteins that often interact with ACs and PDEs so that PKA activities can be regulated in a defined spatiotemporal fashion; and lastly, PKA often cross-talks with other signaling pathways that are responsive to cellular growth and metabolic changes, including mTORC and AMPK.

### 3.1. Allosteric Activation of PKA

The molecular mechanisms of PKA activation lie in the structure of its subunits. Since the first atomic structure of the mouse catalytic subunit PKA Cα in complex with a 20-amino acid substrate analog was solved [89], structures of various individual subunits and holoenzymes have been revealed using both X-ray crystallography and solution neutron scattering [20]. Both the R and C subunits of PKA are modular proteins containing various domains. The R subunits of PKA are composed of N-terminal docking/dimerization domain (the D/D domain), followed by a linker region containing a pseudo-substrate peptide (the inhibitory sequence, or IS) and two consecutive cyclic nucleotide-binding domains (the CNB-A and CNB-B domains) [90,91]. The C subunits of PKA are smaller than R subunits in size and appear as a bi-lobal globule separated by a shallow groove [92]. PKA subunits share high degree of homology at the level of amino acid sequence, although non-consensus residues exist in different regions. These differences create PKA subtypes with varied three-dimensional topologies that confer the specificity and efficiency of cooperative cAMP binding and activation. For example, both type I and type II holoenzymes undergo allosteric conformational changes upon the binding of cAMP to the R subunits. However, the C subunits are compacted in type I enzymes, whereas they are more open in type II enzymes, presumably due to their differential interactions with R subunits [93,94]. RIα is an extended Y-shaped protein, whereas RIβ is more globular. Although the overall architecture of α and β regulatory subunits are similar, considerable differences exist between them, causing their varied biochemical properties [95]. In consistence with the biochemical analyses, RIα possesses a higher affinity for cAMP than RII subunits, suggesting that type I PKA is potentially activatable at lower cAMP concentration [96,97]. The formation of the RIα-containing holoenzyme requires Mg^2+^-ATP which acts as a negative orthosteric modulator, whereas RIIβ-containing holoenzyme formation does not, leaving no ATP present in the type II holoenzyme. This also places the type II holoenzyme in a less stable transient state [98]. Other structural differences include the IS that interacts with the catalytic cleft of C subunit. The IS of RII contains a serine in its pseudo-substrate sequence, whereas RI contains alanine at the same position [99]. This feature fits well for the auto-phosphorylation of RII and the open architecture of the C subunit in the type I holoenzyme [100]. The autophosphorylation of RII has been shown to be a pre-requisite for type II holoenzyme to activate [101]. These intrinsic structural differences make R and C subunits behave differently during the PKA inactivation/activation cycle and implicate that PKA subtypes are activated through different modes. Accordingly, RIα is more responsive to lower cAMP concentration and should be quicker in re-assembly, and is essential for the PKA system in maintaining appropriate basal control of the enzyme activity in cells, whereas RIIα is usually tethered to microdomains within cells (such as plasma membrane) and only activatable by higher cAMP concentration generated locally; however, it can endure a longer activation period.

### 3.2. Tissue-Specific Expression and Functions of PKA Subunits

One way to regulate PKA signaling is to restrict the expression of its subunits, so that the functional PKA subtypes are present in different cell types in a spatiotemporal manner. Early studies suggested that different tissues in mammals contain differentially expressed PKA subtypes [102]. For example, whereas α-type PKA subunits are expressed more ubiquitously, RIβ and RIIβ are enriched in brain and adipocyte, respectively [103,104,105,106]. The catalytic subunits of PKA exhibit similar expression patterns [107,108]. Furthermore, PKA subtypes are differentially expressed in various brain regions of the central nervous system [109]. Due to their varied protein-interacting properties, the subcellular distribution of R subunits also varies. RIα is found to be more diffusible in the cytoplasm of cells as a soluble protein, whereas RIIα is usually tethered to cellular membranous or cytoskeletal systems, making it more insoluble biochemically [110,111,112]. The dynamic expression of PKA subunits was exemplified in mammals during germline development [83]. Investigations using rodent models show that germ cells express PKA subunits dynamically along their developmental trajectory. Rat oocytes express both RIα and RIIα before and after germinal vesicle (the nucleus of fully grown oocyte) breakdown [113]. In male gonads, the α-type regulatory subunits (RIα and RIIα) are the main R subunits expressed when spermatogenic cells enter meiosis and differentiate into haploid spermatids, although RIα is also expressed at a modest level in earlier stages [114,115]. Mature sperm contain both α-type R subunits, but RIIα appears to be the dominant species. The expression of β-type subunits is subtler than that of the α subunits in the male germline. Although both RIβ and RIIβ mRNAs were detected in post-meiotic cells, only RIIβ protein was seen in germ cells at low levels, suggesting that α-type PKA is the main subtype in mammalian male germ cells [116]. In addition, male germ cells express a specific splicing variant of the catalytic Cα subunit (Cα2, also known as Cs) that is only expressed after spermatogenic cells enter meiosis and plays important regulatory roles for sperm motility, whereas the canonical Cα subunit is expressed only at earlier stages [117,118]. Due to the alternative splicing, Cα2 lacks an N-terminal 7-aa stretch that probably contains protein myristoylation sites, making it more widely spread in the cytoplasm of spermatogenic cells than the membrane-tethered Cα subunit [23]. In both male and female gonads, the somatic cells surrounding developing germ cells also express PKA subunits, mainly the type II subtypes. Interestingly, Tasken et al. reported that PKA activity induced by cAMP elevation could positively regulate the expression of RIα and RIIα subunits in a feedback fashion, although maybe through different control mechanisms [119]. Whether different subtypes of PKA elicit differences in the same tissue during aging of the animal, and how the dynamic expression and distribution of subunits facilitate the regulation of PKA specificity, require further investigation.

Mouse genetic studies using gene-targeted deletion showed that the specific functional roles of PKA are well complemented by the expression of its subunits. All seven subunits have been analyzed using this approach and the phenotypes elicited by these genetically modified mice revealed differential functional modes of PKA subunits in a tissue and cell-type-specific manner. For example, RIβ^−/−^ mice exhibit brain-specific phenotypes [120]. In these mice, the basal PKA activity in the neuron is not affected; however, once stimulated with cAMP and PKI (PKA inhibitory) peptides, PKA activity in the brain was lower compared to the wild type. Other gene-knock-out mouse models also suggested varied effects of subunits on PKA activities, including that of RIIβ in lipid metabolism in adipocytes [106,121]. Analyses also revealed that the regulation of PKA activity differs due to the composition of PKA holoenzymes and the complementary expression of subunits could be induced when one of the homologous subunits is disrupted. Studies show that deletion of RIα gene had a wide range of defects in multiple tissues at embryonic stage, whereas RIIα gene mutant, on the contrary, did not show deterioration of physiology due to the complementary increase in RIα expression [122,123,124]. To further elucidate the physiological roles of PKA, more precisely controlled conditional gene deletion mouse models that could eliminate PKA subunits in specific tissues at specific developmental stages would be helpful.

### 3.3. AKAPs as the Organizing Center of PKA Signalosome

It is important to regulate the specificity and efficiency of PKA activity so that the enzyme can precisely control the cellular activities in response to a spectrum of external stimuli without aberrant ramifications. Other than expressing PKA subunits in a cell-type-specific manner, cells have achieved this also by confining different subtypes of PKA into defined subcellular compartments so that the enzymes gain access to their potential substrates more precisely and effectively [26,125,126]. The first evidence showing that the cAMP-dependent protein kinase is enriched in particulate fractions of cell homogenates suggested that PKA may be associated with membranous or cytoskeletal systems [2,11]. Others have also found that radiolabeled cAMP could be enriched in rough microsome fractions when cells were fractionated [127]. Using biochemical methods, Theurkauf and others found that the association of PKA with microtubule fractions in brain and heart tissues was mainly due to the interactions of RII subunits with MAP2 (a microtubule associated protein) and the p75 protein, respectively [128,129], leading to the discovery of A-kinase anchoring proteins (AKAPs) [130]. The protein p75 was later named AKAP75, which was the first AKAP family member identified. Accumulating evidence shows that PKA regulatory subunits interact with various AKAPs expressed in different cell types and tissues. Although AKAPs are defined as the interacting proteins of R subunits of PKA, there are only 14 proteins named as AKAP (AKAP1 to AKAP14), while the rest of the proteins interacting with R subunits bear random names. Nonetheless, the AKAP family so far contains over 50 members encoded by at least 40 genes in the mouse genome and this number will likely continue to grow [131,132]. Like PKA subunits, AKAPs facilitate the regulation of PKA activity in a spatiotemporal fashion with a wide range of physiological functions, whose deteriorations lead to diseases including cardiovascular and neuronal diseases [133,134,135,136]. Following initial biochemical analyses, protein structural studies indicate that AKAPs utilize a conserved 14-18-amino acid long amphipathic helix motif to interact with the D/D domain of PKA R subunits [137,138,139,140,141,142]. This conserved motif (i.e., N′-LAXXIAXXIVXXVM-C′) contains hydrophobic residues at every two positions, generating a polar surface in the three-dimensional structure that fits into the hydrophobic cleft formed by the X shaped tetra-helix configuration of D/D domain [131,137]. Based on the protein structure and peptide array studies, similar peptides with preferential residues that make contacts with R subunits were tested and modified for their affinities and specificities with either RI or RII subunits [137,143,144,145,146]. Most AKAPs bind RII with higher K_m_ as they were originally discovered; however, studies have also identified AKAPs that specifically bind RI subunits, including SKIP (Sphingosine kinase interacting protein) and PAP7 (PBR and PKA-associated protein 7) that target RIα specifically to mitochondria [147,148]. A small membrane-tethered protein was also found to tether RIα close to the plasma membrane [149,150]. Naturally, some of the AKAPs are dual-specificity AKAPs that could bind both RI and RII, although with varied affinities, and only elicit preferences for binding of different R subunits under discrete conditions [151,152]. It is currently not clear how cells regulate the differential interactions of AKAPs with R subunits. Besides the amphipathic helix, AKAPs contain less conserved domains and disordered regions among different family members, giving a tremendous flexibility of AKAPs to interact with a variety of protein partners, including membrane receptors, ion channels, signaling molecules, enzymes that counter PKA activities and organellar proteins [126]. The complexity of AKAP structures makes it difficult to analyze their domain–function relationships precisely, as the depletion of AKAPs may interrupt multiple signals and cellular activities. However, genetic studies in mice have provided data to support the structural implications [153,154], especially when AKAPs are expressed or mainly functional in particular tissues [155,156]. It is now well conceived that AKAPs form a platform to bring together signaling molecules and substrate effectors so that PKA activities can be controlled rapidly with precision. The understanding of AKAPs could be enhanced by the acquisition of their full structural properties, which so far are only partially solved [157,158]. In addition, in vivo detection of AKAP activities would be required via the development of more sensitive methods, such as FRET-based reporter systems [159]. This could help to understand how AKAPs regulate their interactions with different R subunits and how they form varied protein complexes that integrate PKA signaling activities.

### 3.4. Non-Canonical Partners of PKA and Cross-Talks of Signal Transduction Pathways

Cyclic AMP is a metabolic product of adenosine tri-phosphate (ATP), which is the main product of respiratory reaction in mitochondria. This implies that PKA signaling is tightly associated with the metabolic status of cells. It was suggested that ATP could act as an allosteric competitor of cAMP for RIα, whereas it facilitates the binding of cAMP to RIIβ [98]. Other sensors of cAMP in the cell, including EPAC (Exchange protein directly activated by cAMP) and CNG (cyclic nucleotide gated ion channel) could also facilitate the regulation of PKA activities by balancing the local concentrations of cAMP. EPACs are guanine nucleotide exchange factors (GEF) for the small G protein RAP [160]. They contain cyclic nucleotide-binding (CNB) domains that autoinhibit their own C-terminal catalytic domain to interact with RAP, which can only be released upon cAMP binding [161]. EPAC2 has been shown to regulate Golgi-derived microtubule during the biogenesis of insulin-containing vesicles in islet β-cells, indicating the role of EPAC during insulin secretion [162]. Other than the regulatory mechanisms mentioned above, PKA activities can be modulated by alternative means. The catalytic subunits of PKA have been proposed to interact with proteins named C-KAPs (C-Kinase Anchoring proteins), which could inhibit the catalytic activity of C subunit as a pseudo-substrate and localize the catalytic subunit in specific subcellular compartments independent of R subunits [163]. Similarly, RIα interacts with proteins other than C subunits to modulate the formation of holoenzymes and activities of PKA, such as P-REX1 (PI3P-dependent Rac exchange factor 1) and Darpp32 (Dopamine and cAMP-regulated phosphoprotein 32) [164,165]. In addition, one line of evidence suggested that, within the holoenzyme, the RIα subunit can form inter-molecular disulfide bonds through N-terminal cystine residues in response to oxidative states in cardiac myocytes independent of β-adrenergic receptor stimulation and cAMP elevation [99], providing an additional level of control over PKA activity.

It is conceivable that the cAMP-dependent protein kinase is intricately linked with other signaling pathways that regulate the growth of cells in response to environmental and cellular metabolic changes, including MAPK, ERK, PKC, Hedgehog, AMPK and mTORC signaling pathways [79,166,167]. In fact, early studies on the functional roles of PKA were mainly focused on its metabolic effects, such as the hormone and nutrient responses in the yeast *Saccharomyces cerevisiae* [168,169,170]. Although less is known about how nutrients stimulate PKA signaling in mammalian cells, lactate has been suggested to bind cell surface GPCR as a signaling molecule in some cells besides its role as an alternative fuel [50,171,172]. The lactate-sensing GPCR (GPR81 or HCAR1) could reduce the level of cAMP in cerebral neocortex and hippocampus and acts as a volume transmitter linking neuronal activity and metabolism [172]. Extensive cross-talks between metabolism, growth and cell division occur [173,174]. PKA and other nutrient-sensing signaling pathways coordinate common actions during these physiological changes which reciprocally regulate PKA activity. Both PKA and mTORC activity were found to be necessary during the heat-induced relaxation of nuclear barrier for extrachromosomal rDNA circles (ERCs), an aging factor that is usually retained within the mother cells [175]. In humans, exercise-induced protein phosphorylation in skeletal muscle encompasses multiple protein kinase pathways, including AMPK, PKA and mTORCs. Among over 1004 phosphorylation sites within 562 proteins, AKAP1 was found to be modified by AMPK under this condition [176]. Interestingly, PKA was found to phosphorylate AMPKα and inhibits it during lipolysis and fatty-acid oxidation [177,178]. Metabolically, PKA has been linked to mitochondria and energy metabolism in cells via either AKAPs targeted to the mitochondria or other signaling pathways [69,179]. PKA phosphorylates MIC60 to regulate PINK1 stability and the recruitment of PARKIN to damaged mitochondria in order to initiate mitophagy [70]. PKA Cβ was found to regulate catecholaminergic activity and energy balance [180]. In addition, several lines of evidence indicate that PKA signaling can regulate nitric oxide signaling during ischemic neovascularization by phosphorylating endothelial nitric oxide synthase [181]. It should be noted that the nutrient- and energy-sensing signaling pathways, including mTORC and AMPK pathways, inevitably interact with the regulatory systems that monitor the proteome in cells, for which the functional roles of PKA remain to be fully understood. In the same vein, when yeast encounter poor nutrient conditions, such as low glucose, both PKA and TORC induce a repression on gametogenic genes through inhibiting the expression of IME1 (inducer of meiotic I), a master transcription factor that regulates meiotic genes, preventing yeast from entering sporulation [182]. During this process, a control of TORC1 level seemed crucial. It appeared that PKA and TORC1 signaling coordinate the initial and steady-state responses of glucose-induced gene expression respectively [183]. Using model systems, research in the past has accumulated vast information on how cells regulate gene expression using PKA signaling in response to environmental changes, including the direct modulation of protein synthesis.

## 4. Regulation of Translation Mediated by PKA Signaling

One of the most studied effects of PKA is its ability to modulate gene expression. This can be achieved through the phosphorylation of proteins involved in DNA-binding activities, including epigenetic and transcriptional factors. Histone lysine demethylases and deacetylases are among the first nuclear proteins found to be modified by PKA, affecting chromatin structure for the epigenetic regulation of gene expression [86,184]. The phosphorylation of transcription factors could have widespread effects since it often changes the expression of numerous genes involved in physiological processes, including hormone biogenesis and metabolism, as exemplified by CREB (cAMP response element-binding protein) and ATF1 (Activating transcription factor 1, another CRE-binding protein) [84,85,185]. CREB can translocate into the nucleus and bind consensus promoter sequence (cAMP response element, or CRE) in the 5′ upstream region of genes upon phosphorylation by PKA [186]. It has been shown that the human genome contains several hundred genes with CRE sequences in their promoter regions. Transcription factors, including FOXO1 and GLI, are phosphorylated by PKA during animal development in response to external signals. FOXO1 plays a key role in glucose homeostasis, cell proliferation and differentiation in response to insulin and glucagon. It was found that PKA could phosphorylate FOXO1 at sites similar to those used by PI3K/AKT, suggesting a shared FOXO1-inhibiting function [187]. Phosphorylation of GLI by PKA can cause its cytoplasmic retention and proteolytic degradation, preventing its transcriptional activity that antagonizes Hedgehog signaling during cellular differentiation [188,189]. Following fertilization, the condensation and rearrangement of parental chromosomes are regulated differently. The condensation of maternal chromatin requires AKAP95, suggesting an active role of PKA signaling during maternal nuclear reprogramming [190]. In mouse male germ cells, an orthologue of CREB, named CREM (cAMP response element modulator) has been shown to regulate post-meiotic gene expression, although for a brief period of time [191,192]. PKA modulates the activity of CREM through several co-factors. Phosphorylated KIF17b (by PKA) shuttles ACT (activator of CREM transcription) out of the nucleus, providing a feedback inhibition of PKA on meiotic transcription [193,194], whereas MIWI, a piRNA-binding protein, could interact with phosphorylated KIF-17b and facilitate the export and localization of mRNAs into the chromatoid body (CB), the RNA–protein complex of haploid spermatids [195].

Proteins are the basic functional units of cells. The generation of appropriate protein constituents and the maintenance of homeostasis of functional proteins are thus important for cells to acquire proper identities and perform physiological functions during development, growth and aging. Environmental cues and cellular metabolic programs that reflect conditions under which cells proliferate and function are thus intricately interwoven with protein synthesis. Accumulating studies have shown that cellular proteomes at any given time are subjected to dynamic regulation at multiple levels of translational regulatory networks, including the expression of ribosomal proteins, the assembly/disassembly of translation machinery, the process of translation, as well as the modifications of mRNAs and RNA-binding proteins (RBPs). PKA signaling participates in the regulation of protein synthesis through directly modifying translation factors and RBPs. Schmidt et al. showed that ribosomal proteins might be substrates of PKA in rat brain in the early 70s [196]. In fact, the regulation of translation was among the first functional roles of PKA to be discovered during the study of hormonal and nutritional effects in yeast [197]. Much of what we know about the translational regulation by PKA comes from studies of several model systems, such as yeast and metabolically active mammalian cells, including neurons and germ cells.

### 4.1. Regulation of Translation by PKA Signaling in Yeast

Yeast adjust their life cycle of growth, sexual reproduction and aging in response to nutrient availability, hormone stimulation and other environmental cues, including pH and temperature [173,198,199]. The steady-state growth of yeast requires sufficient nutrients, such as carbon sources, amino acids and fatty acids. However, when there is a shortage of nutrients, for example, glucose depletion, yeast can enter a dormant growth phase, reducing the synthesis of cellular components and growth rate in order to survive until the nutrient supply returns. As a sexually reproducing organism, yeast cells also undergo differentiation in order to generate gametes for mating in response to pheromones [169]. The quick adaptation to the living environment in yeast is mainly due to the nutrient-sensing systems they possess that are intricately linked to their cellular metabolic status [174,200]. Multiple kinase-mediated signaling pathways, including MAPK, RAS/PKA, mTORC and AMPK, participate in the regulation of cellular growth in yeast [182,201]. Although how glucose is sensed and interpreted by yeast remains to be fully characterized, both glucose-sensing GPCR and intracellular RAS GTPase could be activated by glucose, which in turn activate AC to produce cAMP and induce PKA [170].

**Figure 2:**
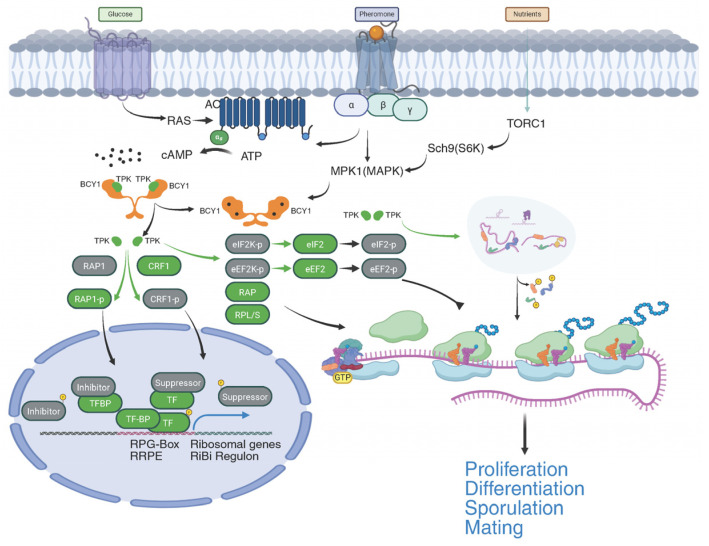
Protein synthesis regulated by PKA signaling in yeast. In response to extracellular stimuli, cell surface proteins, including glucose transporters or G-protein coupled receptors (GPCRs), transmit signals to cortical G proteins and adenylate cyclase to generate local cAMP, which subsequently activates PKA. Activated PKA elicits multiple effects through phosphorylated substrate proteins, including transcription factors, RNA-binding proteins and translation regulators, that facilitate changes in gene expression of translation machinery and translatability of mRNAs. See main text for details. Figure created with BioRender.com (TG29RGLRMJ).

PKA participates in the regulation of the yeast proteome using mechanisms at multiple levels, including transcription, translation and post-translational modifications of various translation regulators. Early studies show that the transcription of genes encoding ribosomal proteins (RPs) decreases once temperature becomes restrictive or glucose is withdrawn from the culture medium [202,203,204]. Analysis of the transcriptional regulation in response to changes of carbon source uncovered consensus upstream activation sequences in the promoter regions of RP genes (the UAS_RPG_ or RPG-box) [88,205]. It was further shown that these upstream regulatory sequences bound by a trans-activating factor TUF1 (translation upstream factor 1), also known as RAP1 (repressor activator protein 1) or GRF1 (general regulatory factor 1), are carried by almost all RP genes in yeast [206,207,208]. These conserved cis-elements synchronize the transcription of RP genes when ribosome biogenesis is in demand and allow yeast to quickly respond to environmental cues. Interestingly, RAP1 could also interact with telomeric sequences, leaving an intriguing connection between protein synthesis, telomere maintenance and aging [209]. The transcriptional programs of RP genes in yeast are further augmented by transcription factors responsive to alternative signals such as SFP1, a zinc-finger-containing transcription factor activated by stress conditions, and co-factors including histone acetylase ESA1 [210,211]. Multiplexed regulatory protein complexes formed at RP gene promoter region elicit either positive or negative effects on RP gene expression. For example, RAP1 can either activate or repress RP gene expression when combined with GCR1 or RIF1 (RAP1-interacting factor 1), respectively [212,213], whereas FHL1 (Forkhead-like 1) regulates RP gene expression either positively with IFH1 (Interacting with forkhead-1) or negatively with CRF1 (Co-repressor with FHL1) [214,215,216]. Multiple kinase-mediated signaling pathways relay their effects on RP gene expression in the midst via the post-translational modification of transcriptional regulators, linking environmental changes to RP gene expression. It was found that phosphorylation of RAP1 by PKA enables its import into nucleus and interaction with promoter of RP genes [217], whereas CRF1 can be activated by YAK kinase (YAK1 in yeast), which can be phosphorylated and suppressed by PKA [214]. A recent study further showed that introns of mRNAs in yeast help to counter starvation by enhancing the repression of RP genes [218]. Thus, the expression of RP genes and the translation machinery are coordinated systematically by PKA via master transcriptional regulators in order to ensure the efficient changes in protein synthesis. The synchronized regulation also occurs in non-ribosomal genes that contain consensus elements, including RRPE (ribosomal RNA processing element) and PAC (RNA polymerases A and C) motifs, which are collectively termed RIBI (ribosome biogenesis) regulon, with several key components under the regulation of nutrient-sensing signaling, including PKA and TORC [204,219]. It was shown that PKA and TORC1 phosphorylate and inactivate transcriptional repressors Dot6 and Tod6 that bind PAC to activate non-RP genes of the RIBI regulon in response to carbon- or nitrogen-sensitive conditions, respectively [220] (Figure 2).

While the transcriptional regulation could have a global and long-lasting effect on cells, regulation at the translational level offers fast flexibility and specificity. It was first observed that the depletion of a carbon source, namely glucose or fructose, could cause a rapid decrease (within about 5–10 min) in the content of polysomes in yeast. The reduction in active translation could be reversed once glucose was added back to yeast culture, indicating that the translational control is one of the first responses during nutrient depletion [221], although these changes are likely gene-specific [222,223]. Direct post-translational modifications of ribosomal proteins by PKA have been observed for both small and large subunits, including RPS10 which is phosphorylated when yeast undergo steady-state growth [224]. PKA substrates also include ribosome-associating proteins (RAPs) that closely monitor the translational activity of ribosomes. During the analysis of the nutrient effect on the growth of yeast *Pichia pastoris*, it was found that PKA phosphorylates a 40-KD RAP to protect cells against vanadate toxicity [225]. Both PKA and SNF1 (the yeast homolog of AMPK) phosphorylate STM (another RAP), whose activity impedes elongation and facilitates the change in programmed ribosome frameshift (PRF) in response to glucose depletion [226,227]. In addition, other components of the translation machinery are also subject to PKA regulation. For example, a reduction in PKA activity due to the glucose deprivation was linked to the blocking of nuclear re-entry of transfer RNA (tRNA) export receptors through an unknown mechanism, reducing the level of cytoplasmic tRNAs and translation activity [228].

Studies in yeast also identified modifications of translation initiation and elongation factors by PKA activity when concentrations of glucose in the environment change. Phosphorylation of eIF2α (eukaryotic initiation factor 2 subunit α) keeps eIF2 complexes from the 5′-cap of mRNAs and halts the initiation of translation. The phosphorylation of eIF2α is carried out by eIF2α kinase (eIF2K) which in yeast is modified by PKA. Addition of glucose activates PKA which helps to release the inhibitory effect of eIF2α phosphorylation via eIF2K. Dietary restriction was shown to decrease the abundance of 60S large subunits in cells, which could induce the expression of GCN4 (general control of amino-acid biosynthesis 4) [229], a well-known transcription factor that is induced by the restriction of amino acids in yeast [230]. Interestingly, GCN4 is a substrate of GCN2, a mammalian eIF2α kinase paralog, which itself can be regulated at translational level through its 5′-uORFs of mRNA when translation of other cellular mRNAs is repressed by PKA [231]. Thus, PKA could modulate translation factors with varied effects on translation under defined circumstances, of which the efficiency and specificity remain to be fully understood.

RBPs interact with mRNAs and modulate their translatability. Cells maintain mRNAs in RNA granules, the cytoplasmic foci containing ribonucleo–protein complexes (RNPs), such as P-bodies and stress granules, in an untranslatable state and release them upon stimulation for translation. RBPs that mediate the assembly and disassembly of RNA granules regulate the translation efficiency of associated mRNAs through these phase transition states [232]. It was shown that PKA activity regulates the formation of P-bodies in yeast through the phosphorylation of the scaffolding protein PAT1 of RNA granules [233]. Extra RNP foci are often formed in yeast during the stress response, coupling to the diminished PKA activity. It was found that phosphorylation of PAT1 at Ser456/457 inhibits the aggregation of the protein and thus the formation of P-bodies when PKA activity is stimulated [234]. PKA also facilitates the localized translation of proteins that are necessary for cellular growth by influencing the localization of cellular mRNAs. It was shown that PKA promotes the mRNAs of HXT2 (hexose transporter 2) to localize into the newly formed bud (the daughter cell) and enables the daughter cell to transport more nutrient for growth [235]. During pheromone-induced mating response, the HXT2 mRNA was believed to be restrained in mother cells via interaction with RNA-binding protein SCP160, which had been shown to be a direct partner of GPA1, the Gα subunit of the G protein trimeric complex that is downstream of a GPCR [236]. Intriguingly, subunits of PKA could be directly localized in RNA granules when yeast encounter increasing temperature, a form of growth stress [237]. The changing localizations of PKA subunits between the cytoplasm and RNA granules are isoform-specific, suggesting functional diversity of different PKA subtypes [237]. In the same vein, studies in mammalian cells found that PKA-RIα could facilitate local cAMP concentration by undergoing liquid–liquid phase separation (LLPS), providing a regional control of PKA activity [238,239,240].

The nutrient-sensing system in yeast includes multiple kinase-mediated signal transduction pathways, including the pheromone-stimulated MAPK pathway, amino acid and nitrogen-sensing TORC pathway, the energy-sensing AMPK pathway, as well as the glucose-sensing RAS/PKA signaling pathway [241]. Each of them appears to take on a specific route of regulation; however, interactions occur when yeast receive complex environmental cues. It is not surprising to see that the energy status of cells modulates AMPK signaling that is sensitive to the AMP/ATP ratio, which could also have effects on TORC and PKA signaling [242]. In this regard, SCH9 (the yeast homolog of mammalian S6K, a substrate of TORC) could phosphorylate MPK (MAP kinase) and inhibits the latter’s kinase activity. BCY1 (homolog to mammalian PKA RIα) phosphorylation by MPK appears to be required for its association with TPK (homolog to mammalian PKA Cα), and now dissociates from TPK [243]. Other than responding to different nutrient conditions, the differences between PKA- and TORC-mediated cellular growth control remain to be defined. A recent study indicates that PKA and TORC may regulate a subset of proteins at different stages during the nutrient-sensing process [183]. Thus, studies from yeast indicate that the regulation of protein synthesis by PKA signaling occurs at multiple steps during mRNA metabolism, translation initiation, elongation, and the biogenesis and modification of ribosomes.

### 4.2. Regulation of Translation by PKA Signaling in Neurons

In response to growth factors, nutrients or neurotransmitters, neuronal cells sustain tremendous prepubertal development and long-term post-mitotic cell growth and maintenance during adulthood. Both pre-synaptic boutons and post-synaptic dendritic spines can adjust their size and activity in an activity-dependent manner, which is supported by local protein synthesis [244,245,246]. The continuous modeling of neuronal morphology is important for the development and functions of neurons, facilitated by nutrient supplies from surrounding glial cells. It was shown that stimulated neurons could use glucose as the energy source through glycolysis, temporarily surpassing oxidative metabolism [247]. However, others also suggested that neuronal cells take up lactate as the alternative fuel since the animal brain is encased within a blood–brain barrier (BBB) [50]. One functional role of lactate provided by glial cells, including astrocytes, is to signal the neurons to either grow or differentiate. This is partly accomplished through an L-lactate-specific cell surface receptor, that was first discovered in adipocytes for its anti-lipolysis function [172,248,249]. A recent study provided a direct link between lactate signaling and protein synthesis in cultured cerebral slices [250]. In addition, lactate was found to trigger neuronal gene expression through an NMDA receptor agonizing mechanism via downstream ERK signal [251].

**Figure 3:**
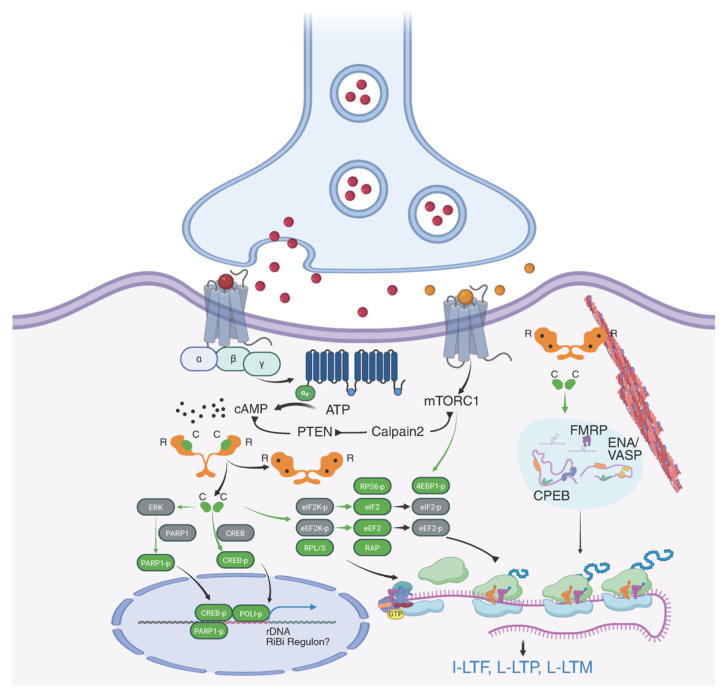
Protein synthesis regulated by PKA signaling in neurons. Similar to yeast, neurons receive signals from adjacent dendrites at synapses or extracellular environment and transmit them through GPCR or surface receptors to elevate local cAMP concentration inside cells, which in turn activates PKA. Although it is not clear whether neurons contain RIBI regulon, rDNA clusters can be activated by phosphorylated transcription factors, e.g., PARP1 and CREB. Both RNA-binding proteins and translation machinery components can also be phosphorylated by activated PKA in order to modulate local protein synthesis in neurons. See main text for details. Figure created with BioRender.com (ZE29RGMQHT).

PKA signaling plays an essential role during neurite outgrowth, growth cone tuning and synaptic plasticity [252,253,254,255]. Although not completely understood, cAMP-mediated signaling regulates gene transcription and the synthesis of proteins at synapses through various mechanisms, including modifications of translation machinery and RBPs [256,257,258] (Figure 3). The activation of the PKA/ERK signaling pathway phosphorylates the PARP1 protein, causing it to translocate from the cytoplasm into the nucleoli. PARP1 is a DNA-binding protein that can re-model the structure of chromatin where ribosomal genes reside, leading to the activation of rDNA transcription. It was found that, in *Aplysia* neurons and mouse hippocampal slices, neuronal activity stimulates the nuclear–cytoplasmic translocation of PARP1, enabling rDNA expression that is required for long-term facilitation [259,260]. Part of BDNF (Brain-Derived Neurotropic Factor) function during neurite outgrowth is to activate RNA polymerase I, the RNA polymerase for the transcription of rDNAs and genes encoding transfer RNAs, in an ERK1/2-dependent manner, although its interaction with PKA signaling remains to be established [261]. It is not clear whether there is a RIBI regulon at work in neuronal cells like in yeast.

PKA could affect translation in neurons through directly modifying the translation machinery. Ribosomes isolated from the rat cerebral cortex contain phosphorylated components of both small and large subunits, including RPS2, RPS6, RPS10, RPL6, RPL13 and RPL29. Some of the ribosomal proteins, such as RPS3A, RPS5, RPL14 and RPL19 could only be phosphorylated by PKA when not assembled into ribosomes [262]. One possibility is that the phosphorylation states of different ribosomal proteins vary when they are either assembled into ribosomes or in the free cytoplasmic pool, which may affect the biogenesis of functional ribosomes or participate in the regulation of translation efficiency. Similarly, it was found that ribosomes from the Chick embryonic brain had tightly associated kinase activities, of which PKA activity increased with development while casein kinase activity decreased [263]. Although the functional significance of RP phosphorylation requires further investigation, it is clear that kinase activities influence protein synthesis via translation initiation and elongation factors in neurons [264]. In neurons from *Aplysia*, stimulation of intermediate-term facilitation with spaced serotonin treatments induces PKA to phosphorylate and inhibit eEF2K, the kinase that phosphorylates and inhibits the translocation activity of eEF2α during translation elongation. The eEF2α activity and protein translation are sustained when neurons are treated with serotonin [265]. Upon BDNF stimulation, mTORC1 signaling was activated in neurites from Sprague Dawley rats, partly through the degradation of mTORC1 inhibitors by Calpain2 which also targets PTEN to regulate the level of cAMP [266,267]. Both RPS6K and eIF4EBP1 can be phosphorylated by mTORC1, linking signaling interplay directly to the protein translation machinery during neurite outgrowth.

RBPs facilitate the translation of bound mRNAs and play important roles in neuronal development and function, either under physiological conditions or in pathological settings, such as neurodegenerative diseases in humans. Among the PKA substrates that are discovered in neurons, the cytoplasmic poly-A element-binding protein (CPEB) is among the first ones known to mediate PKA functions [268]. A recent study suggested that FMRP, the RBP that affects the fate of numerous mRNAs in neurons [269,270], could modulate PKA activity and its interaction with AKAPs to control actin assembly during learning and memory [271]. Both biochemical and mouse genetics studies have provided strong evidence that fragile X syndrome is caused by mutations that occur in FMRP. The transport of neuronal mRNAs to their correct localizations is a pre-requisite for activity-dependent translation regulation. RBPs play important roles during this process and maintaining the translatability of localized mRNAs as well, mainly by forming ribonucleo–protein complexes. In this regard, the discovery of the functional roles of Ena/VASP family proteins in neurons helped to fill the gap between the targeted transportation and translation of neuronal mRNAs [272]. In neurons, MENA (mammalian Enabled) was found to facilitate the localization of neuronal mRNAs and formation of mRNPs, while maintaining their F-actin assembly activity [273]. In the embryonic rat brain and in vitro cultured neurites, BDNF induced dissociation of MENA with neuronal mRNAs and facilitated protein synthesis. The multi-domain protein MENA, which was first found in migrating fibroblasts, fulfills its actin- and mRNA-modifying activities through direct or indirect interactions with various partners [274]. In a Y2H screen, it was found that MENA could interact with the RIα subunit in haploid male germ cells [275], providing MENA as a potential link between PKA activity and the modulation of protein synthesis and neurite morphology [276].

Interestingly, the regulatory circuitry of PKA signaling during activity-dependent changes in neuronal synapses includes PKA regulatory subunits. Various isoforms of PKA subunits apparently have differential roles during the regulation of neuronal growth and synaptic plasticity. It was suggested that type II PKA interacts with cytoskeletal systems in neuronal growth cones and mediates their turning responses [277]. However, PKA RIα was also found to interact with α/β-tubulin in *Aplysia* neurons, suggesting its participation in regulating neuronal morphology during long-term facilitation [278]. In *Aplysia*, both RIα and RIIα could be degraded through ubiquitination and proteasome system once PKA was activated [279]. Additional evidence suggested that it was RIα that had been degraded following serotonin-induced synaptic activity, but not RIIα [280,281]. This may help the catalytic subunit of PKA to sustain PKA functions. Studies from gene-knock-out mouse models showed that neuron-enriched RIβ, once mutated, did not cause major changes in PKA activity in neurons probably due to the compensatory expression of RIα [120]. However, certain aspects of neuronal activity were disrupted in these mice, including defective LTD (long-term depression), motor learning behavior and neuronal gene expression, similar to those of RIIβ and Cβ gene-deleted mice [106,120,282]. Thus, although PKA elicits versatile roles in neurons, the specificity and efficiency underlying its function require further investigation.

### 4.3. Regulation of Translation by PKA Signaling in Mammalian Germ Cells

PKA signaling plays important roles during oocyte growth, meiotic maturation, zygotic gene activation (ZGA) and blastocoel formation in the blastocyst [283,284,285]. The expression of PKA subunits during oogenesis follows a dynamic pattern. In several species, the activity of PKA has been found to be high during oocyte growth and low during meiotic maturation, a process that allows the resumption of meiosis and germinal vesicle breakdown (GVBD) [286,287]. During mouse oocyte growth, cAMP can be generated either within the oocyte upon stimulation of GPCR/Gs or supplied by granulosa cells through cell–cell gap junctions [71,288]. Deletion of the PKA catalytic subunit or AKAPs that are responsible for the proper localizations of PKA in *Drosophila* germline caused destabilization of cortical structure and fusion of nurse cells, leading to oogenesis defects [289,290,291]. The maternal patterning of the oocyte in *Drosophila* is important for early embryogenesis, which is also influenced by PKA localization and activity. For example, the synthesis of the maternal factor Oscar protein is regulated by PKA signaling, in conjunction with the activity of 3′-UTR (untranslated region) of its mRNA [292]. In vertebrates, maintaining the PKA activity appears to be important to keep fully grown oocytes in the meiosis I stage. Reducing PKA activity either through gene silencing or chemical inhibitors promoted oocyte maturation in several species [75,293]. The reduction in PKA activity was believed to alleviate the synthesis of cell cycle inhibitors that keep fully grown oocyte in meiotic arrest. One such factor, the so-called M-phase promoting factor (MPF) complex, once released from a repressive state, is able to activate the meiotic cell cycle into the next (MII) stage. Both Mos and Cyclin B/CDK2 were thought to be part of MPF [286,293]. It was found that ARPP19 (cAMP-regulated protein 19), a potential inhibitor of phosphatase PP2A, was a direct substrate of PKA during the meiotic maturation [294]. Interestingly, different subtypes of PKA may have differential effects on GVBD and oocyte maturation, although the underlying mechanisms are unclear [295,296]. During meiotic maturation, translation activity was found to increase around chromosomal region where mTORC1 signaling appeared to play a role during this process [297,298]. Consistently, mTORC1/4EBP1 activity was found to participate in the meiotic maturation of bovine oocytes [299], although the intrinsic relationship between PKA and mTORC1 signaling during meiotic maturation remains to be determined. PKA was found to regulate ZGA in the early nineties [300]. The expression of a protein complex changed when mouse zygotes completed the first cell division, which could be blocked by inhibiting PKA activity. These so-called transcription-requiring proteins (TRPs) were around 60–70 KD in molecular weight; however, their identities remain unclear [300,301]. Using an in vitro culturing system, Manejwala et al. found that an increased cAMP level induced formation of blastocoel formation, implicating a role for PKA in pre-implantation embryo development [283].

**Figure 4:**
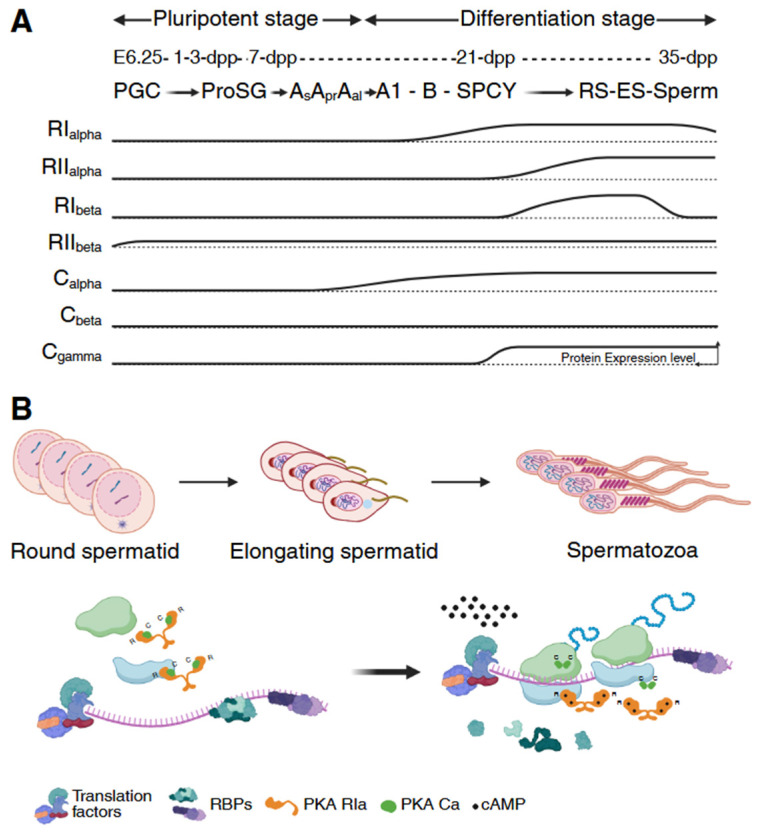
Protein synthesis regulated by PKA signaling in developing mouse spermatids. (**A**): Stage-specific expression of PKA subunits in mouse spermatogenic cells. Mouse spermatogenesis encompasses three consecutive stages, mitosis, meiosis and spermiogenesis. Several isoforms of PKA subunits, including RIα, RIIα, RIβ, Cα and Cβ, are elevated during the late phase of spermatogenesis. The functional roles of various PKA subtypes in spermatogenic cells are not fully understood. (**B**): A model depicting PKA participating in the regulation of protein synthesis during spermiogenesis in mice. Elongating spermatids undergo dramatic cellular morphogenesis, during which nascent proteins are synthesized from pre-stored mRNAs and assembled into subcellular compartments of sperm. It was found that PKA agonist increased levels of nascent protein in transiently cultured elongating spermatids, consistent with the finding that RIα and Cα could co-sediment with polysomal fractions of testicular lysates on the sucrose gradient (Xu et al., Biol. Reprod., 2014) [315]. How PKA regulates translation during spermiogenesis remains to be fully characterized. Figure created with BioRender.com (EA29RGN4RY).

Less is known about the roles of PKA during spermatogenesis, where PKA signaling participates several aspects of the development of spermatogenic cells. Where and how the extracellular signals are generated in order to stimulate intracellular changes in cAMP/PKA in spermatogenic cells at different developmental stages is not completely clear. Spermatogenic cells at different developmental stages are compacted in close proximity within the seminiferous epithelium, where the cAMP signals must have spatiotemporal restrictions of activation instead of a less controllable diffusion pattern. It was suggested that hormones including FSH bind to GPCRs in Sertoli cells in order to stimulate the production of cAMP [42,302]. The expression of atypical adhesion molecules CELSR1-3 (Cadherin EGF LAG seven-pass G-type receptors 1, 2 and 3, Celsr1-3) in both Sertoli and germ cells implicates alternative mechanisms that germ cells could apply to transmit cAMP/PKA signaling explicitly via cell–cell interactions [303,304]. As mentioned earlier, PKA subunits are expressed in a spatiotemporal fashion during mammalian spermatogenesis (Figure 4A). The expression of the main regulatory subunits of PKA (RIα and RIIα) elevates once spermatogenic cells enter meiosis and develop into haploid cells (spermatids) [116]. The expression of the catalytic subunit also changes from Cα to the sperm-specific splicing variant Cα2 (or Cs) around the same time [117]. In addition, several studies showed that a soluble form of AC is expressed in post-meiotic round spermatids [47,305]. These suggest that the activity of PKA is fine-tuned during spermatogenesis via specific PKA subtypes. This notion is supported by mouse genetic studies showing that mutations of various PKA subunits elicited specific phenotypes in the male gonad [83]. In addition, lactate, the main energy source of meiotic cells, also provides signaling information for cellular processes [306]. Whether lactate is mainly transported through MCTs (mono-carboxylate transporters) or spermatogenic cells express a lactate-responsive GPCR remains to be explored. PKA signaling regulates the motility of mature sperm [307]. Activation of PKA is a pre-requisite for the tyrosine phosphorylation that is essential for sperm to acquire hyperactivity [308]. PKA substrates in sperm presumably activate tyrosine kinases and protein phosphatases in order to modify proteins that regulate sperm motility, although the underlying mechanisms require further investigation.

Post-meiotic development of haploid spermatids (often referred to as spermiogenesis, the last phase of spermatogenesis) encompasses a two-week long developmental process in mouse. Due to the chromatin condensation, much of the cellular changes during spermiogenesis rely on post-transcriptional and translational regulation [309]. Previous studies have found that post-meiotic spermatogenic cells employ sophisticated regulatory systems to control the synthesis of proteins precisely in the right place at the right time [310]. Hundreds of mRNAs have been found to become translationally active upon entry into the elongating stage of spermiogenesis, while others become repressed through RBPs or degraded [311]. Comparative bioinformatics analyses of a dozen post-meiotic mRNAs from mice identified conserved RNA sequences in both 5′- and 3′-UTRs that could drive the translation of these mRNAs [312]. However, compared to the number of genes that are translationally regulated in spermatids, much more cis-regulatory elements within these mRNAs have yet to be characterized. RBPs often withhold mRNAs in RNA granules when protein synthesis is inhibited. Male germ cells contain various RNA granules that constantly store or degrade mRNAs, exemplified by the chromatoid body (CB) in round spermatids. The CB contains numerous RBPs in complex with mRNAs, the exact compositions of which are yet to be identified [313]. As mentioned previously, an atypical kinesin that drives the export of the transcription factor CREM could enter the CB in response to the cAMP signaling during the early phase of spermiogenesis [193], linking the transcription of post-meiotic messengers to their subsequent translational control. Although how cAMP/PKA signaling regulates both transcription and translation during the subsequent processes are not fully understood, several lines of research indicate that PKA activity regulates protein synthesis during the late phase of spermatid morphogenesis via specific PKA subtypes. In RIα heterozygous 129svJ/C57BL/6 hybrid mice, sperm developed abnormal heads and flagella, leading to decreased motility and male sterility [314]. This haplo-insufficiency is similar to the phenotypes observed in humans with Carney Complex syndrome (CNC), indicating that RIα has an essential role during spermiogenesis. In the same vein, it was found that type I PKA was distributed in the polyribosomal fraction of testis homogenates and the activity of PKA positively regulated protein translation in elongating spermatids [315]. Analyses of the expression and subcellular localizations of PKA subunits also suggested that type I and II PKA subunits are localized in different cytoplasmic domains. RIα is diffusely distributed in the cytoplasm of round and elongating spermatids, whereas RIIα appears to associate with subcortical and peri-nuclear cytoskeletal elements [315]. Their distribution in the cell may be associated with the functional roles of PKA signalosomes formed by AKAPs. Consistent with this, mouse genetics and sperm proteomic studies showed that the disruption of sperm-specific AKAP3 not only affected the formation of subcellular structures, and therefore the morphology of sperm, but also caused the accumulation of protein synthesis machinery in developed sperm [316], indicating the likelihood of active translation along the cellular process in developing spermatids. In addition, a yeast two-hybrid screen using a cDNA library generated from mouse round spermatids found that RIα potentially interacts with numerous proteins, including the aforementioned MENA and RPS15a [275]. It will be of interest to find out whether PKA could regulate local protein synthesis through direct interaction with translation machinery in developing spermatids (Figure 4B). Interestingly, studies in *Drosophila* found that the cytoplasmic cilia (a ciliated structure formed in the posterior cytoplasm of developing spermatids) may support the localized synthesis and addition of flagella components at the distal end, although the regulatory signaling pathways during *Drosophila* spermiogenesis remain to be determined [317].

## 5. Concluding Remarks and Future Questions

As the mediator of external stimuli for cellular growth and development, PKA naturally interconnects cellular proteostasis and metabolism. The versatile functions of PKA can be regulated at multiple levels, including cell surface receptors that receive external signals, effector proteins that modulate the level of secondary messenger cAMP, anchoring proteins that provide spatial control of PKA activities, and myriad substrates. The combinatorial effects of the PKA regulatory networks are often complemented by the tissue-specific expression of its subunits. Understanding how cells achieve the functional specificity of PKA signaling through its complex regulatory network is important for understanding PKA functions in different physiological processes.

The allosteric mechanism of the PKA holoenzyme has served as the paradigm for enzymatic activation since its discovery. Structural studies have revealed the molecular inner workings of the regulatory and catalytic subunits of PKA under various active/inactive states. However, solving the structures of multi-component super-complexes such as the AKAP-mediated PKA signalosome in its native conditions is a challenge due to its complexity. Thus, how the PKA signalosome forms in cells and what the structural bases are for the modulation of specificity and efficacy of PKA signaling remain to be seen. These questions could be better answered by combining biochemistry, high-resolution imaging, in situ proteomics with high spatiotemporal sensitivities and the improved cryo-EM/ET methodologies.

Research has shown that PKA modifies components of translation machineries, including RBPs. Identifying these substrates of PKA and characterizing their functional roles under different biological contexts would help to gain a better understanding of PKA functions. Mechanistic understanding of whether PKA signaling could directly regulate the activities of translation machinery and how it will affect translation efficiency in cells is also lacking. High-throughput technologies, including single-cell ribosomal profiling, integrated comparison of transcriptomes and translatomes, as well as analyses of the 3D dynamics of ribosomes, may help to delve into the molecular details of PKA functionality.

Understanding how PKA signaling interacts with other signal transduction pathways is crucial for assessing animal development under both physiological and pathological conditions. This may be particularly informative when PKA cross-talks with signaling pathways that are responsive to cellular growth conditions and metabolic states. Multiple pathways, including mTORC and AMPK signaling, participate in the regulation of protein synthesis in response to different stimuli during the proliferation and differentiation of cells. How each of these pathways achieves their functional specificity and whether there are intrinsic cellular programs underlying the action of various signaling pathways remain to be defined. Combining genotypic and phenotypic analyses in both in vitro cellular and tissue models will help to systematically dissect their physiological significance. Information gathered through cellular and biochemical studies should be tested in animal models. In this regard, gene editing systems in mice, for example, would be highly valuable in providing etiological clues to human diseases that are linked to PKA signaling.

## Data Availability

No new data were created or analyzed in this study. Data sharing is not applicable to this article.

## Supplementary Materials

The following supporting information can be downloaded at: https://www.mdpi.com/article/10.3390/ijms27156789/s1.
