# Supplementary material for: Regulation of Translation by PKA Signaling Pathway
Source: Int J Mol Sci. 2026 Jul 29;27(15):6789. doi: 10.3390/ijms27156789 (PMC13466552; doi:10.3390/ijms27156789)
Supplement: Supplementary file 1 [file ijms-27-06789-s001.zip › ijms-4112254-supplementary.pdf]

Table S1. Subunits of cAMP-dependent protein kinase.

PKA Subunit	No. of AA	M.W. ca. (mRNA length)	Expression	Main cellular Localizations	PKA activity in KO mice	KO Mouse Phenotypes	References
Prkar1a (RIα)	381	41.91 KD (3.2Kb, 2.9Kb, 1.7Kb)	Ubiquitous	Cytoplasm	Increased baseline PKA activity, reduced PKA activity*	Early embryonic lethality, defects in mesoderm morphogenesis, primitive heart tube formation	Titani, et al., Biochemistry, 1984; Amieux, JBC, 2002
Prkar2a (RIIα)	402	44.22 KD (6Kb, 2.2Kb)	Ubiquitous	Plasma membrane, cytoskeleton	Increased baseline activity in muscle, reduced PKA activity*	Normal growth and healthy	Takio, et al., PNAS, 1982; Burton, PNAS, 1997
Prkar1b (RIβ)	381	41.91 KD (2.6Kb)	Brain, testis, B/T lymphocyte	Cytoplasm	No effect on PKA activity in brain	Defects in LTD, de-potentiation, injury-induced inflammation and pain	Clegg, et al., PNAS, 1988; Brandon, PNAS, 1995
Prkar2b (RIIβ)	416	48.76 KD (3.2Kb, 1.6Kb)	Brain, adipocyte, endocrine tissues	Cytoplasm	Increased baseline activity in adipose, reduced PKA activity*	Lean, resistant to diet-induced obesity (DIO), increased lipolysis, UCP-1 in BAT, reduced insulin, vLDL, neuronal gene expression and motor learning	Jahnsen, et al., JBC, 1986; Cummings, Nature, 1996
Prkaca (Cα)	351	38.61 KD (2.4Kb)	Ubiquitous	Cytoplasm	Dramatic decrease of PKA activity	Post-natal early lethality, growth retardation, defected sperm motility	Ukler, et al., PNAS, 1986; Skalhegg, ME, 2002
Prkacb (Cβ)	338	37.18 KD (4.7Kb)	Brain-enriched	Cytoplasm, plasma membrane, cytoskeleton	Decreased basal PKA activity in brain, unchange d in adipose and heart	Neurological defects, resistant to diet-induced obesity (DIO), dimorphic energy metabolism	Uhler, et al., JBC, 1986; Howe, MCN, 2002
Cγ	343	37.73 KD (2.8Kb, 1.8Kb)	Ubiquitous				Beebe, et al., ME, 1990;
Cα2 (Cs)	343	37.73 KD (1.8Kb)	Testis	Cytoplasm, flagella	Reduced testis PKA activity*, sperm lack both basal and stimulated PKA activities	Male infertile, defect in $HCO3^-$ induced motility and Ca^{2+} entry, normal spermatogenesis and initiation of motility, defective hyperactivation	San Agustin, et al., BOR, 2001; Nolan, et al., PNAS, 2004

*when examined in combination with cAMP or PKI.
